# Simulating the dynamics of targeted capture sequencing with CapSim

**DOI:** 10.1093/bioinformatics/btx691

**Published:** 2017-10-28

**Authors:** Minh Duc Cao, Devika Ganesamoorthy, Chenxi Zhou, Lachlan J M Coin

**Affiliations:** 1 Institute for Molecular Bioscience, The University of Queensland, Brisbane, St Lucia, Australia; 2 Department of Genomics of Common Disease, Imperial College London, London, UK

## Abstract

**Motivation:**

Targeted sequencing using capture probes has become increasingly popular in clinical applications due to its scalability and cost-effectiveness. The approach also allows for higher sequencing coverage of the targeted regions resulting in better analysis statistical power. However, because of the dynamics of the hybridization process, it is difficult to evaluate the efficiency of the probe design prior to the experiments which are time consuming and costly.

**Results:**

We developed CapSim, a software package for simulation of targeted sequencing. Given a genome sequence and a set of probes, CapSim simulates the fragmentation, the dynamics of probe hybridization and the sequencing of the captured fragments on Illumina and PacBio sequencing platforms. The simulated data can be used for evaluating the performance of the analysis pipeline, as well as the efficiency of the probe design. Parameters of the various stages in the sequencing process can also be evaluated in order to optimize the experiments.

**Availability and implementation:**

CapSim is publicly available under BSD license at https://github.com/Devika1/capsim.

**Supplementary information:**

Supplementary data are available at *Bioinformatics* online.

## 1 Introduction

High-throughput sequencing (HTS) has tremendously revolutionized genomic studies for the ability for cost- and time-effective characterization of the complete genetic information of a sample. In many clinical applications, only a panel of actionable regions are the subject for investigation (Bellos *et al.*, 2014; Samorodnitsky *et al.*, 2015). In these analyses, investigators often use a targeted capture sequencing protocol where a pool of synthesized oligonucleotides (probes) are used to selectively capture genomic fragments of interests using hybridization (Gnirke *et al.*, 2009) In an efficient design, only DNA fragments from the targeted loci are sequenced. This allows for deeper sequence coverage compared to whole genome sequencing at a much lower cost and faster time to results, resulting in a scalable approach for use in clinical laboratories.

Computational simulation has been indispensable in developing and benchmarking HTS data analysis tools (Escalona *et al.*, 2016). Simulation data *in silico* are cheaper and faster to produce than real data; they are generated under controlled conditions and can be perfectly characterized. Furthermore, simulation also helps investigators to assess the performance of sequencing protocols, and to optimize the design prior to performing experiments. While numerous simulators are available for whole genome sequencing (Escalona *et al.*, 2016) and targeted exome sequencing (Kim *et al.*, 2013), to the best of our knowledge, there is currently no existing tool, which can simulate the dynamics of the captured process *in silico*. We believe such a tool would be useful for assessing not only the computational analysis pipeline, but also the efficiency of a capture design.

Here, we present CapSim, a software package to meet the need for simulating targeted capture sequencing data. Given a set of probes, CapSim simulates the dynamics of probe hybridization *in silico* to generate a set of fragments to be sequenced. Unlike most existing HTS simulation tools, CapSim emulates all various stages of the sequencing process, including fragmentation, fragment capture and sequencing. CapSim is written in Java and is able to run natively on any computing platform, making it easily accessible to bioinformatics community.

## 2 Materials and methods

The sequencing process starts with the fragmentation of the DNA, CapSim simulates this process by iteratively sampling fragments from the genome sequence with a given length distribution. We model the fragment length using a log-logistic distribution, which was found to provide the best fit to the fragment size distributions from several datasets (See Supplementary Material).

In the next step in targeted capture sequencing, the target probes bind to the fragments by hybridization and the bound fragments are then pulled down by beads, which specifically binds to the DNA fragments hybridized to the capture probes. CapSim simulates this process by mapping the probes to the fragments. To be computationally efficient, CapSim first maps the probes to the genome sequence and once a fragment is sampled from the genome, it uses a greedy algorithm to determine the maximum number of probes that can bind to the fragment. We model the stochastic nature of the capture process in which the probability of a fragment being captured is proportional to the number of probes bound, and is inversely proportional to the length of the fragment. This simulation of the dynamics of the hybridization is shown to generate more realistic captured sequencing data than Wessim Kim *et al.* (2013), the only existing tool for simulating captured sequencing (see Results).

The captured fragments are then subjected to *in silico* sequencing. For Illumina sequencing, CapSim introduces a size distribution of fragments that form clusters for sequencing. It uses a Log-Logistic distribution to sample from fragments simulated from the capture step (see Supplementary Material). These selected fragments are then used for sequencing simulation in which reads are copied from the two ends of the fragments with errors introduced. For PacBio sequencing, CapSim simulates polymerase read length from a given distribution (see Supplementary Material). In simulating the sequencing of a fragment, CapSim alternates copying the two strands with a PacBio error profile, until reaching the polymerase read length.

## 3 Results

We evaluated CapSim by comparing its simulation and real data from capture sequencing on the same set of probes. We used Agilent SureSelect Target Enrichment kit to enrich the targeted regions and performed capture sequencing on NA12878 sample on both Illumina and PacBio sequencing platforms (See Supplementary Material).

**Fig. 1:**
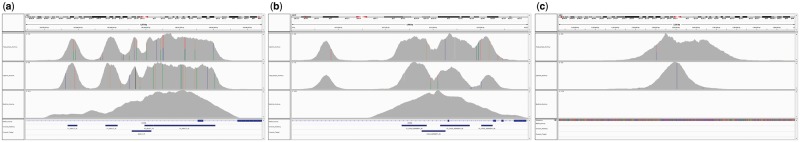
Sequence coverage distribution of Illumina sequencing data from real capture sequencing data on test sample (first panel), capture simulation data from CapSim (second panel) and WesSim (third panel). Position of the capture probes are shown in blue in the bottom capture track panels. (**a**) and (**b**) are sequence coverage in targeted regions and (**c**) is sequence coverage in an off-target region

Sequence coverage distribution between simulation data and real capture sequencing data from Illumina sequencing are shown in Figure 1 for various regions. Coverage distribution from CapSim simulation data closely resembles the real sequencing data. Especially, Figure 1b shows an off-target capture (1 kb upstream of the target region) in test sample which was detected by CapSim simulation data, however Wessim (Kim *et al.*, 2013) did not replicate the sequence coverage distribution of real capture sequencing data and the off-target capture was not detected. CapSim simulation detected 67% of the off-target regions from real capture data, whereas Wessim only detected 8% of the off-target regions (see Supplementary Material). CapSim simulation for PacBio also closely resembles the real sequencing data (see Supplementary Material). Wessim does not support simulation of long read capture sequencing data. 

## 4 Conclusion

In this manuscript, we have introduced CapSim a tool for simulation of targeted capture sequencing on both Illumina and PacBio platforms. Unlike most existing HTS data simulators, CapSim provides parameters for controlling each intermediate stages, allowing users to evaluate the effects of the experiment design before running the experiment (details in GitHub). Once sequencing reads are simulated using CapSim, users can align the reads to the reference genome and assess the performance of the capture design. A customized script to identify probes leading to off-target capture is also provided with CapSim (details in GitHub). This will allow users to identify probes which needs to be removed from the design to reduce the off-target capture to improve their design. We believe CapSim is applicable for all hybridization based capture methods, however the data presented in here was simulated with Agilent XT and more assessment needs to be performed to determine if the simulation data could be comparable for other capture methods.

## Funding

The research is supported by funding from the National Health and Medical Research Council (APP1052303).

*Conflict of Interest*: none declared.

## Supplementary Material

Supplementary Data
