# Supplementary material for: Simulating the dynamics of targeted capture sequencing with CapSim
Source: Bioinformatics. 2017 Oct 28;34(5):873–4. doi: 10.1093/bioinformatics/btx691 (PMC6192212; doi:10.1093/bioinformatics/btx691)
Supplement: Supplementary Data [file btx691_capsim_cao-et-al_supplementary.pdf]

# Simulating the Dynamics of Targeted Capture Sequencing with CapSim – Supplementary information

**Minh Duc Cao[1,†], Devika Ganesamoorthy[1,†], Chenxi Zhou[1], and Lachlan J.M. Coin[1,2,*]**

[1]Institute for Molecular Bioscience, The University of Queensland, Brisbane, St Lucia, QLD 4072, Australia;
[2]Department of Genomics of Common Disease, Imperial College London, London W12 0NN, UK

## Targeted Capture Sequencing

We selected 142 Variable Number of Tandem Repeats (VNTRs) for the targeted sequencing analysis [2]. Agilent SureSelect DNA design was used to design target probes to capture the targeted regions. Target enrichment was performed using SureSelectXT Target Enrichment kit (Agilent) according to the manufacturer's instructions. Sequencing libraries from enriched DNA were prepared using SureSelectXT Target Enrichment for Illumina Paired-end Sequencing (Agilent) and PacBio Template Prep kit (PacBio) for Illumina sequencing and PacBio sequencing, respectively.

## Data sets and processing

We used three sequencing data sets to analyse the properties of sequencing fragment size distribution. These data sets include two capture sequencing data sets (targeting 142 VNTR loci) for the human sample NA12878, sequenced on Illumina MiSeq and PacBio [2] and WGS of NA12878 sample on PacBio [5].

For the in house data sets, the distributions of fragments size prior to sequencing were obtained by analysing the products on Agilent BioAnalyzer 2100. We also calculated the sizes of the fragments that are sequenced in both Illumina and PacBio platforms. In particular, for Illumina data, we mapped the paired-end sequencing data to the respective reference genomes using bwa-mem [4], and extracted the inferred insert size from the bam files. For PacBio, we examined the subread names, and grouped together the subreads from the same polymerase read. To estimate the size of the fragment giving rise to the subreads, we used the average length of all subreads from the fragment, except for the last subread which might not represent the whole fragment. The length of the PacBio polymerase read was determined by the sum of all subreads as well as the adapters.

## Modelling DNA fragment size and PacBio polymerase read length

We fitted a large number of probability distributions (over 80 distributions currently implemented in the Python Numpy V1.18 library) to the histograms of fragment size of the above mentioned data sets. Supp. Fig. 1 presents the fit of the most fitted distributions, including Log-Logistic (Fisk), Burr, Log-Normal, Log-Gamma, Levy, Mielke and Johnson SB distributions on four data sets. Of these distributions, we found distributions in the Log-Logistic family (Fisk, Burr, Mielke and Johnson SB) were consistently among the best fitted distributions, though Mielke and Johnson SB distributions seemed to be not robust in the presence of short fragments. We performed the same analysis for the histograms of polymerase read lengths of the PacBio sequencing data, and also found Fisk distribution fitted the best (Figure 2).

The Log-Logistic distribution has been used for modeling of the distribution of wealth or income in economics [1] and for survival analysis. It is a heavy-tailed distribution that

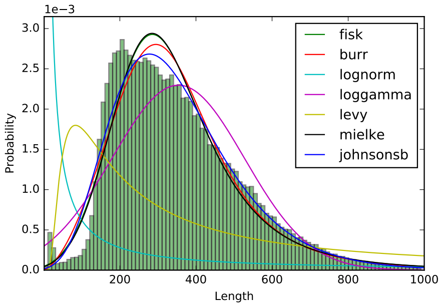


(a) Capture Illumina sequencing of sample NA12878

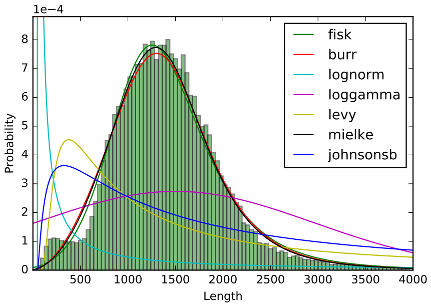


(b) Capture PacBio sequencing of sample NA12878

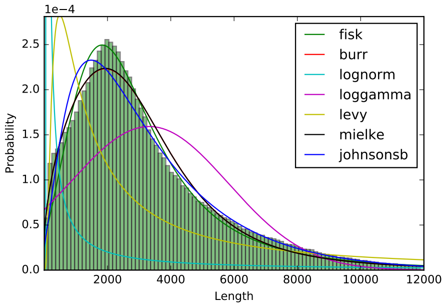


(c) Whole genome PacBio sequencing of sample NA12878

Supp. Fig. 1: Fragment size distributions obtained from four data sets. Various probability distributions were fitted to the fragment size distributions

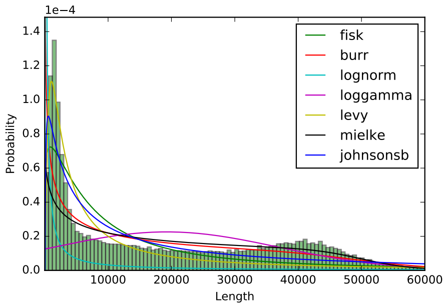


(a) Capture PacBio sequencing of sample NA12878

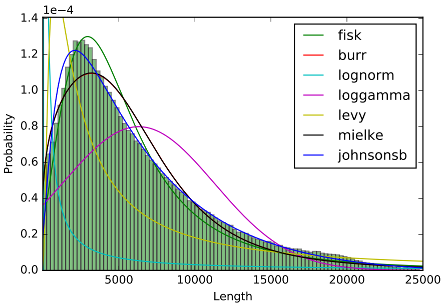


(b) Whole genome PacBio sequencing of sample NA12878

Supp. Fig. 2: Distributions of polymerase read lengths in three PacBio data sets.

explains the good fit to the distributions of fragment size and long read length distributions.

## Effects of capture and sequencing on fragment size

Supp. Fig. 3 and 4 present the fragment size distributions at various stages of sequencing on Illumina and Pacbio platforms respectively. Note that the fragments here included the 60 base adaptors from both ends. We noticed the fragments obtained from capturing tend to be slightly shorter than those prior to capture. On the capture Illumina data set, the

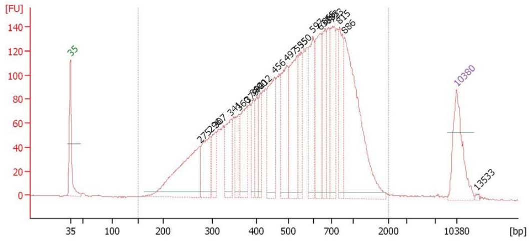


(a) Pre-capture fragment size

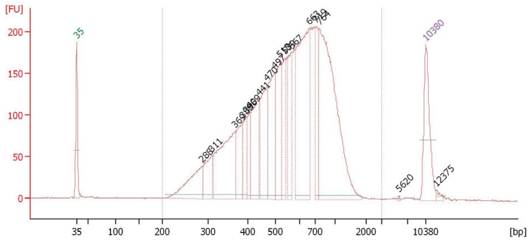


(b) Post-capture fragment size

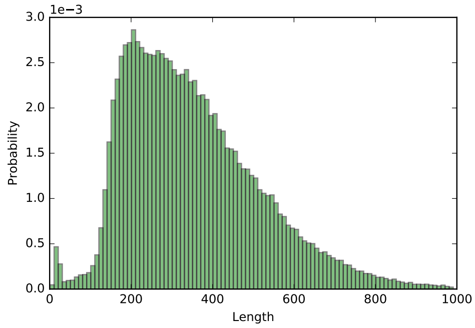


(c) Sequenced fragment size

Supp. Fig. 3: Fragment size distributions of the capture Illumina sequencing of the human NA12878 sample at different stages: a) pre-capture, b) post-capture and c) after sequencing.

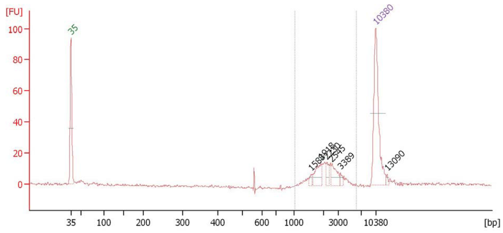


(a) Pre-capture fragment size

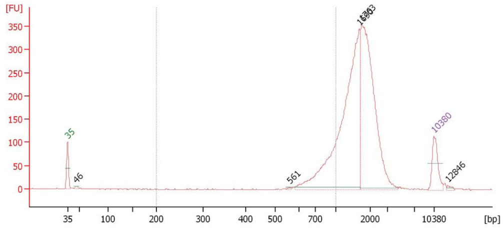


(b) Post-capture fragment size

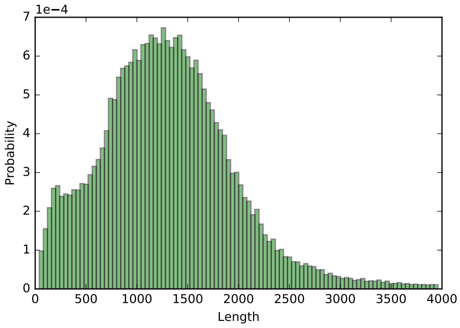


(c) Sequenced fragment size

Supp. Fig. 4: Fragment size distributions of the capture PacBio sequencing of the human NA12878 sample at different stages: a) pre-capture, b) post-capture and c) after sequencing.

pre-capture fragment size distribution peaked at nearly 800 bp, but that from post-capture fragment peaked at over 700 bp (Supp. Fig. 3a and 3b). Similarly, for the PacBio data sets, the modes pre-capture and post-capture fragment size distributions were about 2000 bp and 1700 bp, respectively (Supp. Fig. 4a and 4b). This suggests the capture process has the tendency to select shorter fragments.

We also noticed the fragments that were sequenced on both platforms were even shorter. The mode of the fragment size (without adaptors) distribution from Illumina data was just over 200 bp (Supp. Fig. 3). This is equivalent to a mode of 320 base-pairs for fragment with adapters, which is markedly shorter than the post-capture fragment library (mode of 700 base-pairs). This is explained by the clustering process preferentially amplifies shorter libraries in a mixture of fragments [1]. The reduction of fragment sizes from captured library to sequenced

[1] Nextera® Library Validation and Cluster Density Optimization, Illumina, URL:http://www.illumina.com/documents/products/technotes/technote_nextera_library_validation.pdf, Assessed 16 Sep 2016

fragments on the PacBio is less dramatic, from about to 1.7kb to 1.5kb (Supp. Fig. 4). We attribute this to that smaller fragments are more likely to be selected to translocate through the wells in the PacBio SMRT cell.

## Implementation of CapSim

In a capture protocol, only the fragments that are hybridized with probes (captured) are retained for the sequencing step, while the others are washed away. Instead of simulating the fragmentation of the whole genomes where most fragments are not captured, CapSim marks the regions on the genome that can be captured. With this, CapSim only needs to sample fragments from these regions to significantly reduce running time. We examined the real captured data and found that there were fragments captured with the hybridisation of only 30 bp to a probe. We therefore used bowtie2 [3] with permissive parameters and allowing multiple position alignments (–local –very-sensitive-local –mp 8 –rdg 10,8 –rfg 10,8 -k 10000) to align probes to the genome sequence. The regions which have at least one probe aligning to are marked for sampling captured fragments.

As described previously, the clustering of fragments to the Illumina flowcell favours fragments library in the range of 100-200 bp. For a long fragment library, most of fragments will be not be clustered for sequencing, and hence the simulation of these fragments would be wasted. Instead, from the distribution of fragment size specified by the users for fragmentation (fragmentation fragment) and the fragment distribution preferred by the clustering process (clustering distribution), CapSim uses a Monte-Carlo technique to generate a distribution of fragments that actually clustered and sequenced. Briefly, CapSim iteratively samples from the fragmentation distribution. These samples are then selected or rejected following the clustering distribution. The selected samples are then used to generate the fragment size distribution of fragments that are actually clustered for sequencing. By generating this distribution before the simulation of fragmentation, CapSim can avoid the unnecessary simulation of the fragments that are not used for sequencing.

## Performance of CapSim

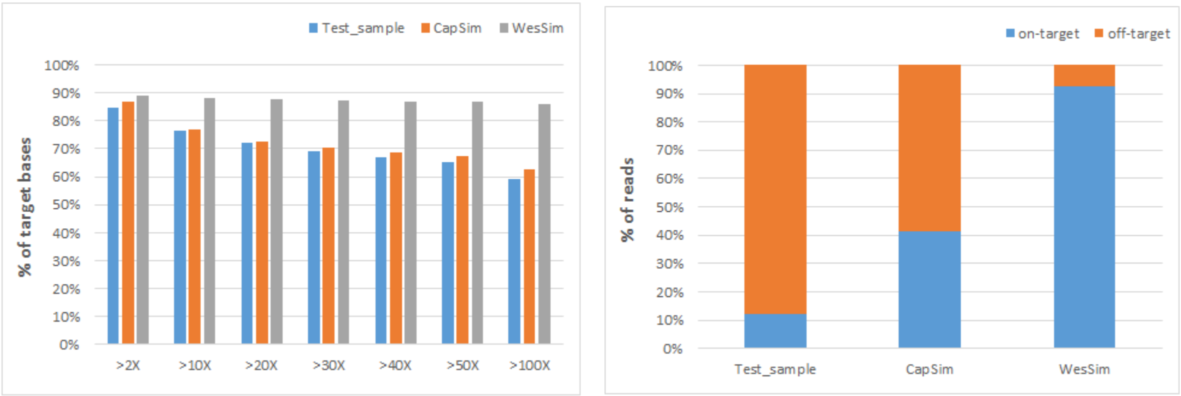


(a) (b)

Supp. Fig. 5: Performance of CapSim: (a)% of bases at specified coverage in targeted regions and (b) % of on-target and off-target regions for real capture data and simulated data from CapSim and WesSim.

We used Illumina capture sequencing data from NA12878 sample to assess the performance of the CapSim simulation and WesSim simulation. All 3 datasets were mapped to hg19

reference using bwa-mem. We used Picard to calculate the capture metrics (Fig. 5). Percentage of target bases at specified coverage was similar to real capture data and CapSim simulation data, atleast 70% of on-target bases had greater than 30x coverage. Only 12% of on-target capture was achieved with real capture sequencing data, and this is due to the capture design of repetitive regions. Low on-target rate is likely due to the alignment issues with repetitive sequences. Nevertheless, the exact alignment settings were used for all the datasets to avoid any bias in alignment. CapSim simulation data achieved 41% on-target, whereas WesSim achieved 93% on-target. Furthermore, figure. 6 shows the off-target regions which were detected by CapSim, but not by WesSim. These results demonstrate that CapSim simulation produced comparable results to real capture sequencing data than WesSim.

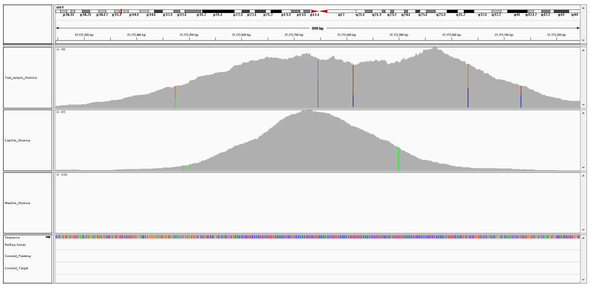

(a)

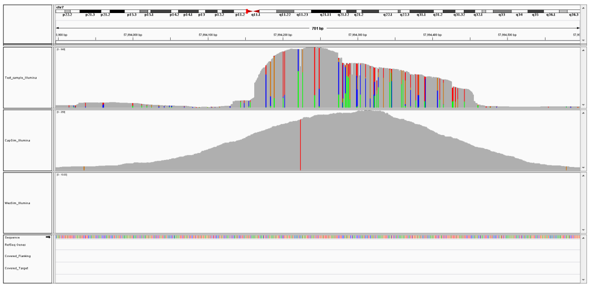

(b)

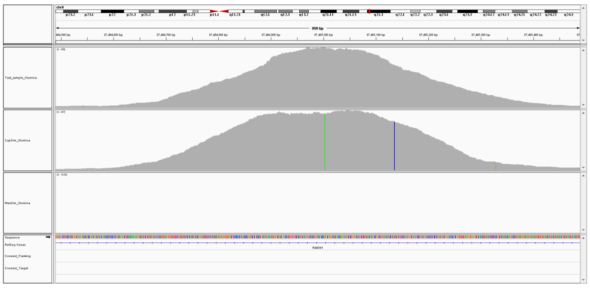

(c)

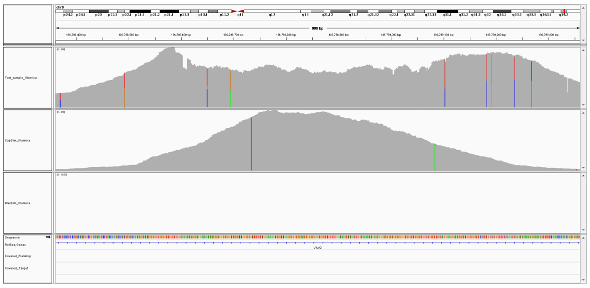

(d)

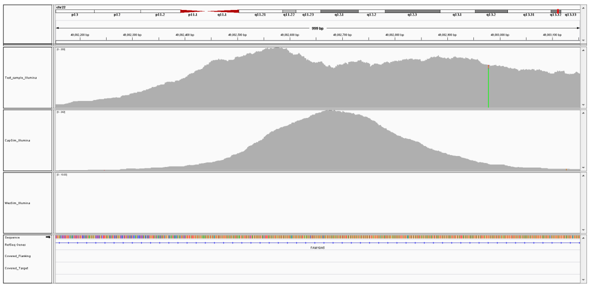

(e)

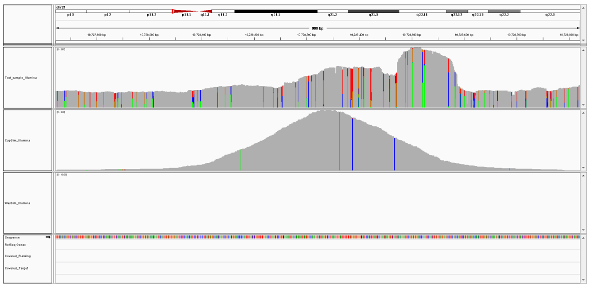

(f)

Supp. Fig. 6: Sequence coverage distribution of Illumina sequencing data from real capture sequencing data on test sample (first panel), capture simulation data from CapSim (second panel) and WesSim (third panel). Position of the capture probes are shown in blue in the bottom capture track panels. (a) - (f) are different regions in the genome, which has off-target capture in real capture data in test sample, which was also successfully detected by CapSim.

CapSim was also used to generate PacBio sequencing data and the performance of CapSim was comparable to real capture PacBio sequencing data. WesSim does not support simulation of PacBio sequencing data, therefore there wasn't any comparable tools to assess the

performance of CapSim for PacBio sequencing data

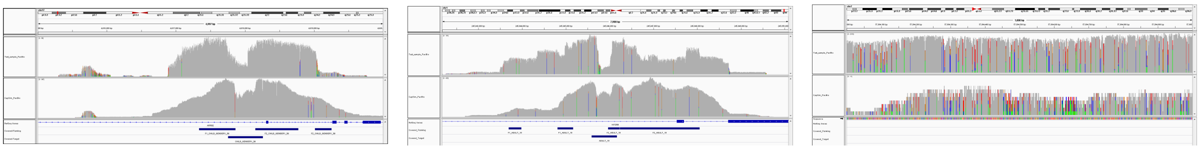

(a) (b) (c)

Supp. Fig. 7: Sequence coverage distribution of PacBio sequencing data from real capture sequencing data on test sample (first panel) and capture simulation data from CapSim (second panel). Position of the capture probes are shown in blue in the bottom capture track panels. (a) - (c) are different regions in the genome which were successfully simulated by CapSim.

## References


[1] Fisk, P. R. (1961). The Graduation of Income Distributions. *Econometrica*, **29**(2), 171.

[2] Ganesamoorthy, D., Cao, M. D., Duarte, T., Chen, W. C., and Coin, L. J. M. (2017). Genotyping tandem repeats by high throughput sequencing. *Under Preparation*.

[3] Langmead, B. and Salzberg, S. L. (2012). Fast Gapped-Read Alignment with {B}owtie 2. *Nature Methods*, **9**(4), 357–359.

[4] Li, H. (2013). Aligning sequence reads, clone sequences and assembly contigs with BWA-MEM. page 3.

[5] Pendleton, M., Sebra, R., Pang, A. W. C., Ummat, A., Franzen, O., Rausch, T., Stütz, A. M., Stedman, W., Anantharaman, T., Hastie, A., Dai, H., Fritz, M. H.-Y., Cao, H., Cohain, A., Deikus, G., Durrett, R. E., Blanchard, S. C., Altman, R., Chin, C.-S., Guo, Y., Paxinos, E. E., Korbel, J. O., Darnell, R. B., McCombie, W. R., Kwok, P.-Y., Mason, C. E., Schadt, E. E., and Bashir, A. (2015). Assembly and diploid architecture of an individual human genome via single-molecule technologies. *Nature Methods*, **12**(8), 780–786.
